# Limits of Free Speech

**DOI:** 10.1007/s11406-016-9752-5

**Published:** 2017-04-05

**Authors:** Lord Bhikhu Parekh

**Affiliations:** 1 Department of Politics and International Affairs, University of Hull, Cottingham Rd, Hull, HU6 7RX UK; 2 Department of Politics and International Affairs, University of Westminster, 309 Regent St, London, W1B 2UW UK; 3 House of Lords, SW1 OPW, London, UK

**Keywords:** Free speech, Legal ban, Hate speech, United States

## Abstract

Free speech is a great value and forms the life blood of a civilised society. It is however, one of several values and may sometimes come into conflict with them. In those cases it may need to be restricted. Hate speech is one such case and the author argues that it can and should be prohibited.

Professor Cohen-Almagor has written an excellent book, both timely and carefully argued. I agree with much of it and commend him for saying it. My disagreements are relatively minor and at two levels. First, while sharing his defence of free speech, I would like to strengthen it and give it a different philosophical orientation. Secondly, I would like to narrow and sharpen the concept of hate speech, my main concern, and give additional reasons for banning it to a greater degree than he does.

The importance of Free speech can hardly be exaggerated. It is the indispensable basis of free thought and critical self-consciousness. When it is denied or severely curtailed, the human capacity to think, and all that is distinctive to human beings, is undermined. At the most basic level, thought is inseparable from speech. Speech is objectified thought, and thought is articulated in speech and represents a form of speaking to oneself. When individuals externalize and express their thoughts and give them a worldly reality, they systematize and impose a measure of order on them, become clearer about their content, create a vitally necessary space or distance between themselves and their thoughts and feelings, and are able to reflect on them critically.

Free speech is the basis of a meaningful human life. It is through the medium of speech that individuals disclose themselves, appear before others, are recognized and affirmed by them, and acquire a sense of who they are. They communicate their thoughts and feelings, build bonds, construct shared memories, and create and sustain a rich and varied individual and collective life. When speech is drastically curtailed and subjected to all manner of constraints, human relations remain shallow and fragile, are marred by ignorance, misunderstanding, dishonesty, and falsehoods, and lack transparency and trust.

Free speech is just as vital in political life. It subjects citizens’ beliefs and opinions to critical scrutiny, assists the formation of an informed public opinion and collective will, provides the only effective check on the government, enables citizens to create a vibrant civil society, and in general ensures an easy and constant flow of ideas among citizens and between them and the government. Free speech is just as crucial to intellectual inquiries. It enables us to challenge inherited dogmas, critically examine different bodies of ideas, expand our range of intellectual and moral sympathy, and to secure a firmer grasp of the grounds of our beliefs and practices.

Although free speech is an important value, so are others such as human dignity, equality, freedom to live without harassment and intimidation, social harmony, mutual respect, and protection of one’s good name and honour. Because these values conflict, either inherently or in particular contexts, they need to be balanced. Although we may rightly privilege some of them over others either in general or in particular contexts, none can be so absolutized as always to trump others. Even social harmony and national security, which are vital to the very existence of society, do not override the demand of human dignity and dree speech, which is why we rightly refuse to sacrifice the latter even when faced with terrorist attacks. We want security and peace but not at all cost, and we do not wish to live in a society that can only be maintained by locking up everyone who arouses our suspicion or by subjecting convicted criminals to human and degrading punishment or torture. Every value makes claims that limit those of others, and every right is limited in its content and scope by other rights. This is as true of the right to free speech as of others, which is why it is subject to limits in all societies. The important question is whether prohibition of the expression and promotion of hatred should be one of these. Here I would be inclined to go further than Prof. Cohen-Almagor.

Hate speech stigmatizes the target group by ascribing to it qualities widely regarded as highly undesirable and presenting it as legitimate object of hostility. The target group cannot be trusted to be a loyal member of society which would be better off without it. Society may eliminate or expel it or discriminate against it and tolerate it as unavoidable evil. All forms of hate speech encourage and justify discrimination and breathe the spirit of violence.

Hate speech is objectionable for both intrinsic and instrumental reasons, for what it is or manifests and for what it does. It lowers the tone of public debate, coarsens the society’s moral sensibility, and weakens the culture of mutual respect that lies at the heart of a good society. It views members of the target group as an enemy within, refuses to accept them as legitimate and equal members of society, lowers their social standing, and in these and other ways subverts the very basis of a shared life. It creates barriers of mistrust and hostility between individuals and groups, plants fears, obstructs normal relations between them, and in general exercises a corrosive influence on the conduct of collective life. Hate speech also violates the dignity of the members of the target group by stigmatizing them, denying their capacity to live as responsible members of society, and ignoring their individuality by reducing them to uniform specimens of the relevant racial, ethnic, or religious group. When hate speech is allowed unrestricted expression, its target group is right to conclude that the state either shares the underlying sentiments or does not consider their dignity, self-respect, and well-being important enough to warrant action. In either case, the state forfeits its legitimacy in their eyes and weakens its claim to their loyalty.

Harte speech is also unacceptable because of its likely long-term consequences. It encourages a climate in which, over time, some groups come to be demonized and their discriminatory treatment is accepted as normal. Vicious and widespread hatred of a group does not spring up overnight. It builds up slowly, through isolated utterances and actions, each perhaps trivial individually but all cumulatively capable of coarsening the community’s sensibility. The violence implicit in hate speech then comes to the fore, initially in isolated incidents but gradually gathering a momentum of its own. If anything can be *said about* a group of persons with impunity, anything can also be done to it. A moral climate is created in which harm done to it is seen as right and proper and does not arouse a sense of outrage. It is not surprising that, with some exceptions, Europeans, who witnessed the rise of Fascism and Nazism and watched the case with which these movements created a racist climate should be some of the strongest advocates of the need for a timely ban on hate speech.

The argument that speech may be restricted only when there is an “imminent danger” of violence fails to probe further the idea of imminent danger. No action occurs in a historical vacuum, and every action produces consequences, not inherently but against a particular background. If a group came into existence urging people to kill all elderly parents or all beautiful women, its intended audience as well as well as its target group would think it was mad or joking and dismiss its utterances without a moment’s thought. But if it expressed similar sentiments about blacks, Jews, or gays, its statements could provoke those so inclined or their long-suffering victims, to resort to violence because of the deeply rooted prejudices against these groups. Imminent danger occurs against the background of, and is imminent because of, the prevailing social climate, and consistency demands that we concentrate our efforts not only on fighting the immediate source of danger, but also its long terms causes including the climate leading up to it.

Because hate speech is in unacceptable for these and related reasons, it has no place in a decent society and deserves to be discouraged. The difficult question is whether it should not merely be discouraged by moral and social pressure but also prohibited by law. Although law must be our last resort, its intervention in the print media or the internet cannot be ruled out for several important reasons. First assuming meaningful levels of enforcement and compliance, prohibition would reduce or eliminate speech that causes very real harm to the targets of such speech.

Second, prohibition legitimizes the state in the eyes of all citizens, reassures the currently targeted group as well as other members of society – for every one of them can under certain circumstances be a target – that the state values them all equally and is committed to protecting their fundamental interests. Third, such a law lays down norms of civility and sends out clear messages concerning what is or is not an acceptable way of talking about and treating other members of society. Being a collective and public statement of the community’s moral identity and guiding values, the law affirms and enforces these values, has a symbolic and educational significance, and helps shape the collective ethos. Finally, proscription of hate speech plays an important role in preventing political mobilization of hostility against particular groups. This is especially true if the limits are enacted before hate-based organizations have built up powerful networks and support and before their rhetoric has coarsened public stability.

It is argued that evil ideas are best defeated by subjecting them to a critical scrutiny and confronting them with better ideas, and that the answer to hate speech is not less but more speech. This argument, to which Prof. Cohen – Almagor shows some sympathy, makes a valid point but exaggerates it. It is true that respect for fellow human beings requires us to engage critically with their misguided but sincerely held beliefs, and that it is more effective in the long run to refute the basis of these beliefs than to supress their public expressions. There are, however, limits to this approach.

The market place of ideas, on whose competitive scrutiny and fairness this argument relies, is not neutral and does not provide level playing fields. It has biases and operates against the background of prevailing prejudices. When racist, anti-Semitic, and xenophobic beliefs are an integral part of a society’s culture, they appear self-evident, commonsensical, obvious and enjoy a built-in advantage over their opposites. Indeed the latter rarely get a hearing, and if they do, they tend to be dismissed out of hand. Furthermore, a fair competition between ideas requires that they all enjoy equal access to the marketplace, including the popular media and other agencies through which they are communicated. This is rarely the case.

Even assuming that the market is or can be made neutral and equally accessible to all bodies of ideas, it is naïve to imagine that false ideas will always lose out in their battle with true ones. Ideas do not operate in a social vacuum. They are bound up with interests, the prevailing structure of power, and so on, and the victory often goes to those that enjoy the patronage of powerful groups or prey on people’s fears and anxieties. Even as far as material products are concerned, competition does not ensure that quality triumphs over cheap and poorly made products. There is no reason to expect a different outcome at the level of ideas. This is not to deny the importance of marketplace of ideas, but rather to argue that, like the market in general, it needs to be subjected to certain regulatory controls, this is what the ban on hate speech does.

In this context I am a little troubled by Prof. Cohen - Almagor’s heavy reliance on the Millian Principle of interfering with an individual only when his or her utterances and actions threaten to cause harm to others, and his concomitant demand that we should show clearly who is harmed, how much, and so on, the kind of demand we make of a plaintiff in a court of law. First, it puts the onus not on the speaker but on his target group, and in so doing places the latter at disadvantage. It assumes that free speech is a privileged value and that anyone aiming to limit it must make a case for doing so. This is a questionable assumption and morally biased.

Second, harm is not easy to define, identify and prove. A relentless decline in the society’s climate of mutual respect is a case of serious harm, but it does not point to specific individuals. Again, racist remarks might undermine the self-confidence of a section of the target group, but lead its other members to overcompensate by excessive concentration on achievements in intellectual, financial and other areas. The latter is harmed because its choices are constrained and heteronomous not free, but it does not appear as a harm. Again, an isolated case of hate speech can be taken in one’s stride. But over time such remark create a climate that might be deeply offensive and unacceptable. At what point did the harm begin and where should we draw the line? Not easy to determine. Professor Cohen-Alamgor is right to insist that exaggerated and ill-focused remarks about harm should not be made the basis of actions on the internet or elsewhere, but the opposite extreme of requiring a fairly precise casual connection between a particular form of hate speech and a particular kind of harm would not do either.

